# Rutin and Hesperidin Alleviate Paclitaxel-Induced Nephrocardiotoxicity in Wistar Rats *via* Suppressing the Oxidative Stress and Enhancing the Antioxidant Defense Mechanisms

**DOI:** 10.1155/2023/5068304

**Published:** 2023-02-22

**Authors:** Yasmine A. Ali, Osama M. Ahmed, Hanan A. Soliman, Mohamed Abdel-Gabbar, M. Al-Dossari, N. S. Abd El-Gawaad, El-Shaymaa El-Nahass, Noha A. Ahmed

**Affiliations:** ^1^Biochemistry Department, Faculty of Science, Beni-Sued University, P.O. Box 62521, Beni-Suef, Egypt; ^2^Physiology Division, Zoology Department, Faculty of Science, Beni-Suef University, P.O. Box 62521, Beni-Suef, Egypt; ^3^Research Center for Advanced Materials Science (RCAMS), King Khalid University, P.O. Box 9004, Abha 61413, Saudi Arabia; ^4^Department of Physics, Faculty of Science, King Khalid University, P.O. Box 9004, Abha 62529, Saudi Arabia; ^5^Department of Pathology, Faculty of Veterinary Medicine, Beni-Suef University, P.O. Box 62521, Beni-Suef, Egypt

## Abstract

Paclitaxel is a primary chemotherapy agent that displays antitumor activity against a variety of solid tumors. However, the clinical effectiveness of the drug is hampered by its nephrotoxic and cardiotoxic side effects. Thus, this investigation aimed at assessing the protective effects of rutin, hesperidin, and their combination to alleviate nephrotoxicity caused by paclitaxel (Taxol), cardiotoxicity in male Wistar rats, as well as oxidative stress. Rutin (10 mg/kg body weight), hesperidin (10 mg/kg body weight), and their mixture were given orally every other day for six weeks. Rats received intraperitoneal injections of paclitaxel twice weekly, on the second and fifth days of the week, at a dose of 2 mg/kg body weight. In paclitaxel-treated rats, the treatment of rutin and hesperidin decreased the elevated serum levels of creatinine, urea, and uric acid, indicating a recovery of kidney functions. The cardiac dysfunction in paclitaxel-treated rats that got rutin and hesperidin treatment also diminished, as shown by a substantial reduction in elevated CK-MB and LDH activity. Following paclitaxel administration, the severity of the kidney and the heart's histopathological findings and lesion scores were markedly decreased by rutin and hesperidin administration. Moreover, these treatments significantly reduced renal and cardiac lipid peroxidation while markedly increased GSH content and SOD and GPx activities. Thus, paclitaxel likely induces toxicity in the kidney and the heart by producing oxidative stress. The treatments likely countered renal and cardiac dysfunction and histopathological changes by suppressing oxidative stress and augmenting the antioxidant defenses. Rutin and hesperidin combination was most efficacious in rescuing renal and cardiac function as well as histological integrity in paclitaxel-administered rats.

## 1. Introduction

Taxanes are natural compounds produced by members of the genus *Taxus*. These compounds are used to treat various cancers and are preliminary therapies for earlier stages of cancer [1–4]. Paclitaxel is a key taxane and an effective antitumor agent [5]. The Food and Drug Administration (FDA) initially approved paclitaxel in 1992 to treat ovarian cancer [6]. Subsequently, the drug was frequently tested for use in the treatment of other cancers, such as breast, prostate, bladder, cervical, and brain [7–10]. However, the clinical use of paclitaxel is considerably restricted because of its limited solubility, recrystallization after dilution, and cosolvent-induced toxicity [11]. Further, cancer cells become resistant to paclitaxel chemotherapy, and the use of the drug causes numerous adverse effects, including neuropathy, cardiac toxicity, and hepatotoxicity [12–14]. Paclitaxel lowers levels of glutathione (GSH) and increases malondialdehyde (MDA) concentrations, suggesting oxidative stress [15], and may have caused renal injury due to induction of oxygen radicals in the reaction, which was a trigger of renal oxidative stress [16]. Furthermore, paclitaxel may cause cardiotoxic effects [17, 18]. According to several studies, different antioxidants can prevent deoxyribonucleic acid (DNA) oxidative stress damage [19–21].

*Citrus* species are an important biological resource from an economic perspective; these plants produce a diversity of phytonutrients and phytochemicals with promising therapeutic properties [22]. Flavonoids exhibit varied bioavailability and biological efficacy. These chemicals may confer health benefits *via*anti-inflammatory, antioxidant, antimicrobial, antiproliferative, proapoptotic, and hormone regulatory properties [23–25]. Combining rutin with other drugs can reduce drug resistance and the side effects of chemotherapy [26]. Rutin eliminated oxidative/nitrosative stress, inflammation, and apoptosis in rat kidney [27]. Also, it significantly inhibited myocardial oxidative insults by modulating ROS levels [28–31]. Hesperidin is a strong candidate phytocompound that displays antimicrobial, anticancer, antioxidant, anti-inflammatory, antidiabetic, and cardiovascular protective properties [32]. The compound also counteracts acute nephrotoxicity *via* antioxidant activity [33]. Further, hesperidin exhibits chemopreventive and chemotherapeutic effects against various carcinomas [34].

Chemotherapeutic drugs such as paclitaxel have several deleterious side effects, including kidney and heart damage, and we intend to reduce these effects by employing plant constituents with antioxidant and anti-inflammatory properties. Thus, this study assessed the renal and cardiac protection by rutin and hesperidin against the toxicity caused by paclitaxel in male Wistar rats. The study also focused on evaluating the functions of the heart and kidneys as well as the histological integrity, architecture, and defenses against oxidative stress and antioxidants.

## 2. Materials and Methods

### 2.1. Chemicals

The formulation vehicle of Cremophor® EL^*∗*^ (CrEL) contains paclitaxel, often known as Taxol or paclitaxel by trade (polyoxyethylated castor oil) (batch number: 7E05628), was obtained from Bristol–Myers Squibb global biopharmaceutical company (Princeton, USA). Rutin (batch number: 501) was obtained from Oxford Laboratory Company (Mumbai, India). From the Sigma-Aldrich Company, hesperidin (lot number: # SLBT3541) was obtained (St. Louis, MO, USA). Creatinine reagent kit (catalog number: M11502c-18) and urea reagent kit (catalog number: M11536c-16) were bought from Biosystem S.A. (Spain), respectively. Uric acid, creatine kinase-MB (CKMB), and lactate dehydrogenase (LDH) reagent kits were purchased from Spin React (Spain); these kits have catalog numbers: MD41001, MD41254, and MX41214, respectively. Chemicals of oxidative stress including trichloroacetic acid (TCA) (batch number: 5O011689) was obtained from PanReac AppliChem ITW Companies (Spain); thiobarbituric acid (TBA) (batch number: L 16A/1916/1212/13) was obtained from Sd Fine Chem Limited (SDFCL) Company (India); 1,1,3,3-tetramethoxypropane or malondialdehyde (MDA) (catalog number: T9889) was obtained from Sigma-Aldrich (MO, USA); metaphosphoric acid (batch number: M 21519) was obtained from ALPHA CHEMIKA Company (India); 5,5'-dithiobis (2-nitrobenzoic acid) (DTNB or Ellman's reagent) (batch number: 40K3652) was obtained from Sigma-Aldrich (MO, USA); GSH (batch number: 3W010085) was obtained from PanReac AppliChem ITW Companies (Spain); pyrogallol (batch number: 1280B251114) was obtained from ResearchLab Company (India). The highest purity and analytical grade reagents were used throughout the investigations.

### 2.2. Physiochemical Properties of Rutin and Hesperidin

Rutin has a molecular weight of 610.5 and the empirical formula C27H30O16. It is a light-yellow crystalline powder that tastes slightly bitter. It has low solubility in water (125 mg/L), while highly soluble in polar solvents, and melts at around 176–178°C.

Hesperidin is a light yellow crystalline powder with the empirical formula C28H34O15 and a molecular weight of 610.6; it is odorless and tasteless. It demonstrated poor pH-independent aqueous solubility; however, it is soluble in formamide and dimethyl formamide at 60°C, slightly dissolve in other polar solvents, and melts at around 258–262°C.

### 2.3. Experimental Animals

Thirty mature male Wistar rats weighing 130–150 g and aging 8–9 weeks were provided as the experimental rats. Rats were taken from the animal house of the National Research Center in Dokki, Giza, Egypt. For avoiding intercompetitive infection, the animals were observed for 15 days prior to the experiment. Rats were maintained in stainless steel-covered, ventilated polypropylene cages that were kept at room temperature (25 ± 5°C) and subjected to 12-hour light/dark cycles every day. Animals received unrestricted water availability but were also nourished with a well-balanced meal *ad libitum* on a daily basis. The Faculty of Science at the University of Beni-Suf in Egypt followed all guidelines and directions issued by the Experimental Animal Ethics Committee (Ethical Approval Number: BSU/FS/2017/8). All efforts have been made to mitigate animal suffering, anxiety, and discomfort.

### 2.4. Experimental Approach

**Figure 1:**
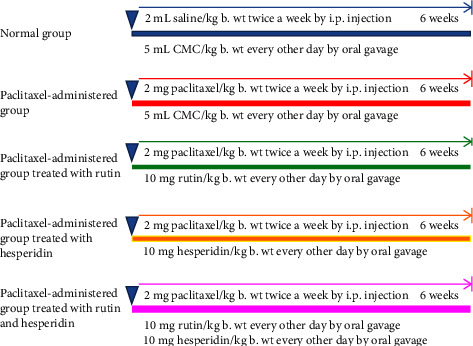
Animal grouping and experimental design.

Male adult Wistar rats had been subdivided into 5 groups in this study (6 rats per group) (Figure 1)

1. Negative control group: this group of rats received 5 mL of 1% carboxymethylcellulose orally (CMC) (vehicle used to dissolve rutin and hesperidin)/kg body weight (b. wt) on an alternate day and 2 mL of isotonic saline (0.9% NaCl) (vehicle in which paclitaxel is dissolved)/kg b. wt twice per week *via* the intraperitoneal (i.p.) route for 6 weeks.
2. Paclitaxel-administered control group: paclitaxel was given to this group of rats at a dose of 2 mg/kg body weight (in 2 mL 0.9% NaCl) by i.p. injection [35] twice a week at the 2^nd^ and 5^th^ days of each week for 6 weeks; every other day an oral equivalent dose from 1% CMC (5 mL/kg body weight) was also administered.
3. Paclitaxel-administered group treated with rutin: this group of rats received paclitaxel as in the paclitaxel-administered control group, as well as rutin at a dose of 10 mg/kg b. wt [36] (dissolved in 5 mL of 1% CMC) taken orally on an alternate day for six weeks.
4. Paclitaxel-administered group treated with hesperidin: Paclitaxel was administered to this group of rats in the same manner as it was to the paclitaxel-treated control group, along with hesperidin, orally twice weekly in a dose of 10 mg/kg body weight [37] (dissolved in 5 mL of 1% CMC).
5. Paclitaxel-administered group treated with rutin as well as hesperidin combination: paclitaxel was delivered to this group of rats in the same manner as it was in the paclitaxel control group, along with rutin and hesperidin at a dose of 10 mg/kg b. wt. (dissolved in 5 mL of 1% CMC) on an alternate day for six weeks.

### 2.5. Blood and Tissue Sampling

Rats were slaughtered under diethyl ether anesthesia [38] after receiving the prescribed dosages for six weeks. Jugular vein blood samples were drawn into gel and clot activating tubes, which were then centrifuged for 15 minutes at 3000 rounds per minute after allowing the clots to form at room temperature (rpm). For biochemical analysis, the sera were rapidly collected, divided into four sections, and kept at 30°C. Following decapitation and dissection, the kidneys and heart were rapidly resected and weighed. Tissue samples from the kidney and heart were removed for biochemical analysis and histopathology analysis. A piece of tissue was excised and transferred to 70% alcohol after being fixed in phosphate buffered formalin (10%) for 24 hours for histology. Using a Teflon homogenizer, 0.5 g of tissue was homogenised in 5 ml of saline (0.9% NaCl) (made by Glas-Col, Terre Haute, USA). The homogenates were then centrifuged for 15 minutes at 3000 rpm, and the supernatants were collected and stored in the refrigerator at −20°C until employed to measure biochemical parameters of antioxidant defenses and oxidative stress markers.

### 2.6. Determination of Serum Kidney Function Parameters

Urea and creatinine levels were detected as previously described, respectively, by Fabiny and Ertingshausen [39] and Tabacco et al. [40]. Uric acid had been measured using a method previously reported by Fossati et al. [41].

### 2.7. Evaluation of Serum Heart Function Parameters

So according to the respective methods belonging to Young [42] and Pesce [43], the CK-MB and LDH activities were assessed.

### 2.8. Assessing the Parameters of the Antioxidant Defense System and Oxidative Stress

Utilizing chemical reagents made in the lab, the heart and kidney oxidative stress and antioxidant defense parameters were evaluated. Lipid peroxidation (LPO) was determined as stated earlier by Preuss et al. [44]. In brief, protein was precipitated by mixing 1 mL of homogenate with 0.15 mL of 76% TCA. Next, 0.35 mL of TBA, a color-enhancing substance, was added to the separated supernatants. After 30 minutes of incubation in a water bath at 80°C, the produced light pink colour was observable at 532 nm. The benchmark was MDA. Based on Beutler et al. [45], the GSH content was evaluated by adding 0.5 mL of DTNB or Ellman's reagent to the homogenate supernatant after protein precipitation (as a color-developing agent). The yellow colour of the samples and GSH standards had been contrasted with a blank at 412 nm. Matkovics et al. [46] procedure was used to determine GPx activity with some modifications. The method works by identifying any remaining GSH and deducting it from the total amount of GSH converted by the enzyme to oxidized glutathione (GSSG). In a Wasserman tube, 350 *μ*l of Tris buffer (pH 7.6), 50 *μ*l of GSH solution (2 mM), and 50 *μ*l of hydrogen peroxide (H_2_O_2_) (3.38 mM) were combined with 50 *μ*l of homogenate supernatant (3.38 mM). The remaining GSH content was then evaluated using the aforementioned procedure for GSH measurement at 430 nm following a 10-minute incubation period. Standard tests were prepared by substituting 50 *μ*l of distilled water for 50 *μ*l of the sample, and a blank test tube was prepared by substituting 100 *μ*l of distilled water for 50 *μ*l each of the sample and GSH solution. The sample's residual GSH concentration may then be detected, and GSH can then be transformed to GSSG to measure the activity of the enzyme. Using the Marklund and Marklund [47] method, SOD activity was evaluated. The suppression of pyrogallol autooxidation by SOD is the basis of the mechanism. Superoxide ions are necessary for the process to take place. The amount of enzyme that results in 50% suppression in extinction changes in comparison to the control after one minute is referred to as one unit of enzyme activity.

### 2.9. Histological Investigations

All animals were immediately decapitated by cervical dislocation and dissection, and their kidneys and hearts were removed. They were subsequently transferred to the pathology department of the faculty of veterinary medicine at Beni-Suef University in Egypt for additional processing, wax blocking, sectioning, and staining with hematoxylin and eosin after being stored in 10% neutral buffered formalin for 24 hours (H&E) [48]. Five random fields were estimated for each section. The number of sections in each group is six. Sections of tissues were examined under light microscopy. The methodology outlined by El-Far et al. [49] was used to determine the histopathological lesion scores. Score scale 0 = normal, 1 ≤ 25%, 2 = 26–50%, 3 = 51–75%, and 4 = 76–100%. The lesions were graded in a blinded manner.

### 2.10. Statistical Analysis

The mean and standard error of the mean were used to reveal all the data SEM ± Mean. The statistical analyses were carried out using the IBM software, USA, Statistical Package for the Social Sciences (SPSS) computer software (version 22). To determine the significance of group means, a one-way analysis of variance (ANOVA) test was conducted. Tukey's posthoc test was then used to compare pair averaged results. Differences were deemed significant at *p* < 0.05 [50]:

(1) $$\begin{array}{c} \%\;change=\frac{Final\;value\;–\;Initial\;value}{initial\;value}x100. \end{array}$$

## 3. Results

### 3.1. Effects of Toxicity Studies on the Kidney Function-Related Serum Parameters

**Figure 2:**
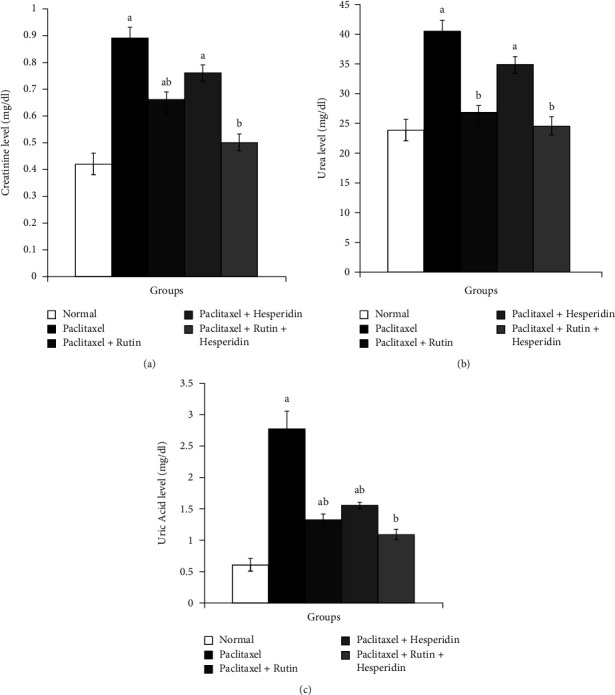
Graphs show the effects of rutin and hesperidin on, serum creatinine (a), urea (b), and uric acid (c) levels in paclitaxel-administered group. ^a^*p* < 0.05: significant compared with normal group. ^b^*p* < 0.05: significant compared with paclitaxel-administered group.

Rats receiving paclitaxel intraperitoneally for six weeks revealed a significant rise (*p* < 0.05) in their serum levels of urea, uric acid, and creatinine with respective percentage changes +111.90, +68.94, and + 361.67%, compared with corresponding normal controls. Rutin and its combination with hesperidin significantly restored increased creatinine, urea, and uric acid levels to normal in paclitaxel-induced rat models. Treatment with hesperidin indicated a nonsignificant decrease (*p* ≥ 0.05) in serum creatinine and urea levels but significant improvement in serum uric acid levels. The combination of rutin and hesperidin treatment of paclitaxel-administered rats was the most efficacious in lowering high creatinine, urea, and uric acid levels, with respective percentage decreases −43.82, −38.03, and −60.65% (Figure 2).

### 3.2. Effects of Toxicity Studies on the Heart Function Related to Serum Biomarkers

**Figure 3:**
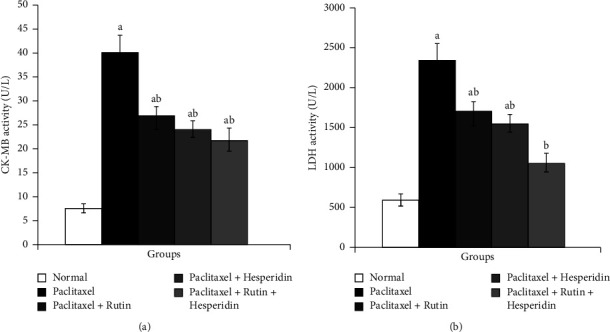
Graphs show the effects of rutin and hesperidin on serum, CK-MB (a) and LDH (b) activities in paclitaxel-administered group. ^a^*p* < 0.05: significant compared with normal group. ^b^*p* < 0.05: significant compared with paclitaxel-administered group.

Rats receiving paclitaxel intraperitoneally for six weeks reported significant alleviation in their serum levels of CK-MB and LDH, with percentage changes of +433.33, +311.85, and +53.31%, respectively, in comparison to the corresponding normal controls. Rutin, hesperidin, and their combination all significantly reduced the increased CK-MB and LDH activity in paclitaxel-induced rats. Treatment with rutin and hesperidin mixture treatmen was most efficacious in reducing high levels of CK-MB and LDH, with percentage decreases of −45.83, −57.54, and −28.92% (Figure 3).

### 3.3. Effects of Toxicity Studies on the Kidney and Heart Oxidative Stress and Antioxidant Defense System Indicators

**Figure 4:**
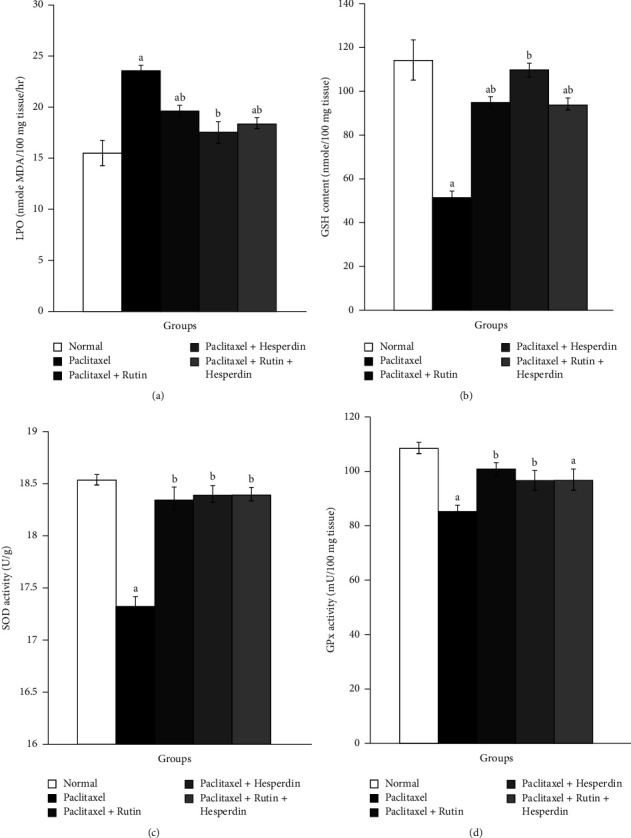
Graphs show the effects of rutin and hesperidin on the kidney LPO (a), GSH (b), SOD (c), and GPx (d) in paclitaxel-administered group. ^a^*p* < 0.05: significant compared with normal group. ^b^*p* < 0.05: significant compared with paclitaxel-administered group.

Following paclitaxel administration, kidney GSH content, SOD, and GPx activities all significantly decreased, while renal LPO significantly increased. Rats that received paclitaxel then treated with rutin, hesperidin, as well as their mixture showed a significant diminished kidney LPO. Hesperidin seemed to be the best in depleting elevated kidney LPO product. Additionally, compared to paclitaxel-administered controls, these treatments dramatically ameliorated decreased kidney GSH content and SOD activity. Only rutin plus hesperidin significantly increased kidney GPx activity (Figure 4).

**Figure 5:**
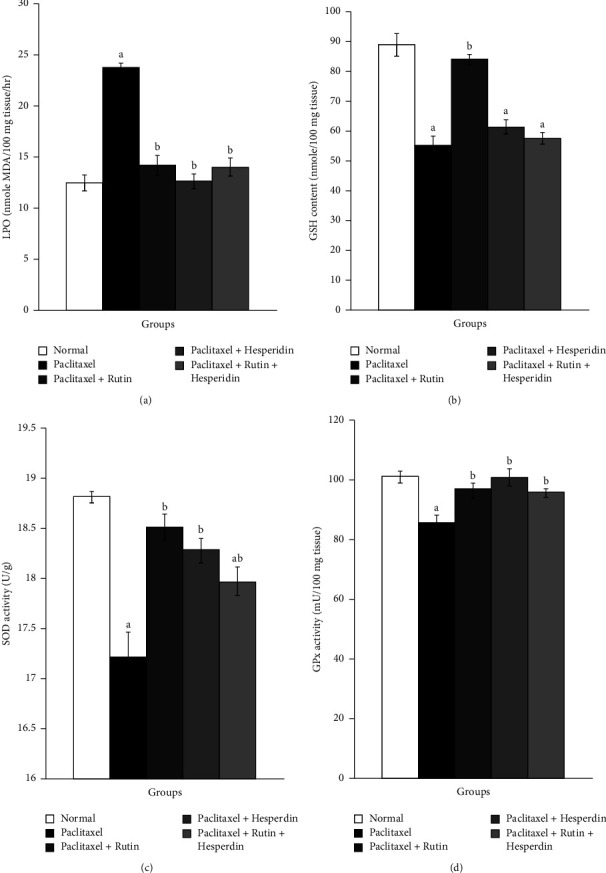
Graphs show the effects of rutin and hesperidin on heart LPO (a), GSH (b), SOD (c), and GPx (d) in paclitaxel-administered group. ^a^*p* < 0.05: significant compared with normal group. ^b^*p* < 0.05: significant compared with paclitaxel-administered group.

Rats receiving paclitaxel for six weeks showed highly significant increases in the heart LPO and significant decreases in the heart GSH content, in addition to SOD and GPx activity. This was similar to what was seen in the kidney. Rats receiving paclitaxel and treated with rutin, hesperidin, and their mixture indicated significantly lower heart LPO. Hesperidin seemed to be the most effective in lowering elevated heart LPO product. Moreover, all three treatments significantly restored decreased heart SOD and GPx activities; only rutin caused a significant rise in cardiac GSH content (Figure 5).

### 3.4. Correlation between Kidney and Heart Function Biomarkers and Oxidative Stress Bio Indicators

The serum creatinine, urea, and uric acid levels showed positive correlation with LPO while they exhibited negative correlation with GSH content and SOD and GPx activities (Table 1).

In addition, the serum CK-MB and LDH activities showed positive correlation with LPO while they exhibited negative correlation with GSH content and SOD and GPx activities (Table 2).

### 3.5. Effects of the Toxicity Studies on the Kidney Histological Changes

**Figure 6:**
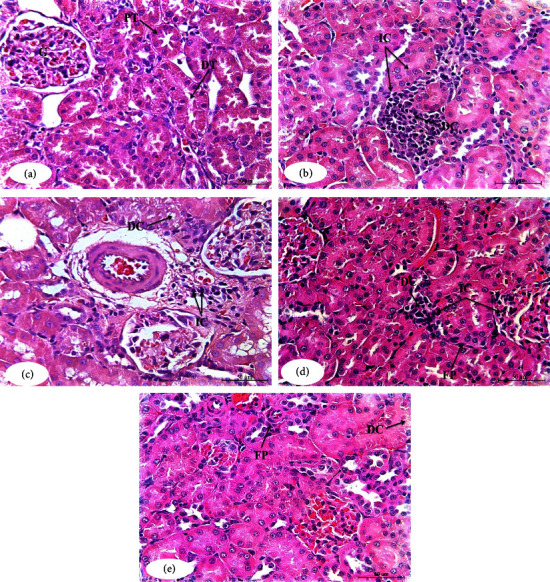
Photomicrographs of the kidney sections of the normal (a), paclitaxel-administered control group (b), and paclitaxel-administered groups treated with rutin (c), hesperidin (d), and their combination (e). G: glomeruli; PT: proximal tubules; DT: distal tubules; DC: degenerative changes; IC: inflammatory cells infiltration; FP: fibroblastic proliferation (H&E; ×400).

Pathological lesions in the kidneys are summarized in Table 3 and illustrated in Figure 6. Normal histological architecture was observed in tissues from normal rats (Figure 6(a)). Paclitaxel-administered animals showed severe pathological lesions, including degenerative changes and nuclear pyknosis of the lining epithelium of the renal tubules associated with glomerulonephrosis in most glomeruli. Focal interstitial nephritis was also observed. Further, congestion was found in the glomerular tuft and interstitial blood capillaries (Figure 6(b)). Paclitaxel/rutin-treated group showed mild to moderate degenerative changes in the renal lining epithelium associated with focal leucocytic infiltration, mainly lymphocytes in the interstitial area (Figure 6(c)). Paclitaxel/hesperidin-treated group suffered from moderate glomerulonephrosis and mild necrosis of lining epithelium of renal tubules (Figure 6(d)). Paclitaxel/rutin/hesperidin-treated group exhibited mild degenerative changes of the glomerular tuft and lining renal epithelium (Figure 6(e)).

### 3.6. Effects of the Toxicity Studies on the Heart Histological Changes

**Figure 7:**
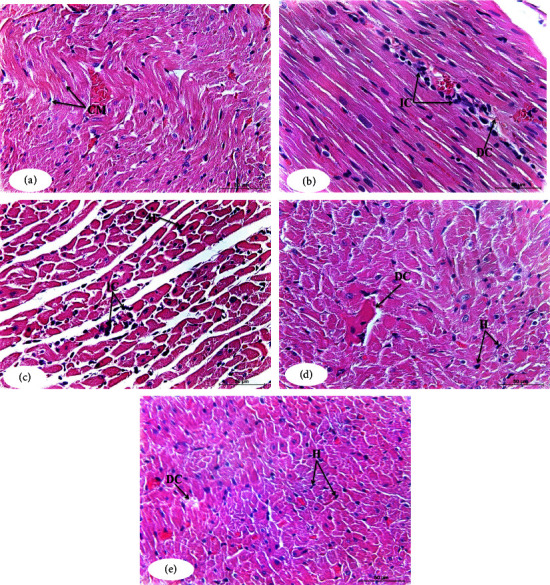
Photomicrographs of the heart sections of the normal (a), paclitaxel-administered control group (b), and paclitaxel-administered groups treated with rutin (c), hesperidin (d), and their combination (e). CM: cardiac muscles; DC: degenerative changes; IC: inflammatory cells infiltration; H: hyalinosis (H&E; ×400).

Detailed pathological lesions are briefly summarized in Table 4 and illustrated in Figure 7. Primary lesions were hyalinosis and lymphocytic myocarditis. Normal histological structure of cardiac muscles was found in normal animals (Figure 7(a)). In contrast, severe hyalinosis associated with focal leucocytic infiltration was seen in paclitaxel-administered control group (Figure 7(b)). Mild lesions were observed in paclitaxel/rutin-treated animals (Figure 7(c)). Paclitaxel/hesperidin-treated group was characterized by an absence of focal lymphocytic myocarditis; however, moderate hyalinosis was seen (Figure 7(d)). Mild degenerative changes of cardiac muscles were seen in tissues from paclitaxel/rutin/hesperidin-treated animals (Figure 7(e)).

## 4. Discussion

Numerous tumours are treated with paclitaxel, including aggressive and metastatic breast cancer, lung cancer, and pancreatic cancer [51]. Unfortunately, paclitaxel therapy can increase the acquired cancer resistance, resulting in chemotherapeutic failure [52]. Paclitaxel may lead to numerous adverse effects on different organs, including the liver, kidney, and heart [53–56]. It promotes oxidative stress, decreases antioxidants, increases liver enzymes, and impairs renal function, which may be due to its mechanism of action and the oxidative stress that it caused [57]. Concurrent use of potent plant antioxidants with chemotherapeutic drugs protects cells and tissues from the harmful effects of free radicals [58].

The nephrotoxicity caused by the intraperitoneal injection of paclitaxel as Taxol was biochemically demonstrated by a significant amelioration in the level of serum creatinine, urea, and uric acid. Paclitaxel inhibits the kidney function and lowers the kidney's ability to remove hazardous metabolic chemicals based on these altered blood levels. These findings are comparable to those of Adikwu et al. [16], who found that paclitaxel deteriorated renal function and caused significant alleviation in blood levels of urea, creatinine, and uric acid, as well as a distortion in normal renal histology. Paclitaxel caused significantly elevated levels of serum creatinine and urea [59, 60]. These alterations in biochemical parameters strongly correlate with several deleterious renal histological changes and lesions, including degenerative changes and nuclear pyknosis of the lining epithelium of the renal tubules, associated with glomerulonephritis, focal interstitial nephritis, and congestion. Other studies have shown similar histological alterations [55, 61–63]. Paclitaxel-induced nephrotoxicity may reflect the alteration and degeneration of glomerular composition and decreased glomerular filtration rate in rats [60]. In our opinion, the kidney histological pathology in renal tissues is due to excessive free radical and ROS production and a reduction of antioxidant defenses. This explanation makes sense given that paclitaxel administration significantly increased renal LPO and significantly decreased renal GSH content of the kidney along with GPx and SOD activity.

In our article, delivering rutin, hesperidin, and their combination of paclitaxel-administered rats successfully reversed kidney pathology as shown by a decrease in serum levels of creatinine, urea, and uric acid as well as ameliorating the kidney histology. These results are consistent with Abou Seif [64] who reported that pretreatment with rutin and hesperidin protects the kidney against nephrotoxicity induced by doxorubicin by improving urea, creatinine, and uric acid serum levels. Rutin provides protection against nephrotoxicity after administration of carfilzomib and has also demonstrated improved histological profiles and ameliorating direct bilirubin, creatinine, and blood urea nitrogen levels [65]. Also, Emam and Madboly [66] showed hesperidin as a potent antioxidant agent that protects the kidney against acetaminophen-induced nephrotoxicity by reversing histopathological changes and reducing blood urea and serum creatinine levels. Likewise, flavonoids such as naringin and naringenin are potent anticancer agents and play a role in the management of various tumors [67].

By increasing serum LDH and CK-MB activity as well as releasing these enzymes from cardiomyocytes into the plasma, the current study discovered that paclitaxel treatment caused cardiotoxicity. These results agree with those of Saad et al. [68], who observed a significant rise in serum CK-MB, LDH, and AST activity, indicating paclitaxel-induced cardiac damage. Notably, Zhang et al. [69] revealed that paclitaxel-induced heart toxicity in normal rats caused significant increases in serum CK-MB levels [70]. These alterations in biochemical parameters correlate with cardiac histopathological findings and lesions that include severe hyalinosis associated with focal lymphocytic infiltration. These results match those of Malekinejad et al. [71], Razzaq et al. [72], and Saad et al. [68], who also found that animals treated with paclitaxel showed severe congestion and necrosis in the heart. Moreover, previous articles have reported that paclitaxel is cardiotoxic [17, 18, 73]. Paclitaxel also was reported to induce apoptosis in cardiac tissue [74]. The heart function and histological integrity may deteriorate from increased oxidative stress, along with a decline in the heart GSH levels, GPx, and SOD activity.

This present article illustrates the ability of rutin and hesperidin to ameliorate elevated CK-MB and LDH activities and normalize cardiac histology in paclitaxel-administered rats. Siti et al. [75] and Xianchu et al. [76] showed that rutin is cardioprotective, and Wang et al. [77] reported that pretreatment with rutin attenuated pirarubicin-induced histopathological alterations and lowered serum LDH and CK-MB activities. An article by Abdel-Raheem and Abdel-Ghany [78] showed that pretreatment with hesperidin protected rats' cardiac tissues from cardiotoxicity caused by doxorubicin *via* reversing histological alteration and reducing serum LDH and CK activities. Pretreatment with hesperidin conserved morphological and ultrastructural architecture of myocardium and reduced LDH and CK-MB activities, supporting a cardioprotective property for hesperidin [79].

Significant increases in the kidney and cardiac LPO, a significant decline in nonenzymatic antioxidant (GSH) concentration, and enzymatic antioxidant (GPx and SOD) activities are all related to detrimental biochemical and histological alterations in the current investigation. Similarly, Ren et al. [80] reported that paclitaxel exposure induced increased ROS and MDA concentrations, while the whole SOD activity declined. These results indicated that paclitaxel administration led to changes in protein expression associated with apoptosis and ROS generation. Also, according to an article, paclitaxel-triggered apoptosis in the renal tubular was linked to decreased mitochondrial membrane potential and a large rise in ROS production. In [81, 82], it was found that a reduction in SOD activity may cause a reduction in superoxide radical ion removal, which may be detrimental to the kidney [64]. An article of Malekinejad et al. [71] showed that paclitaxel administration produced a remarkable increase in the heart LPO and identified the critical role of oxidative and nitrosative stress. Paclitaxel has been recognized to produce reactive oxygen species (ROS) that trigger mitochondrial dysfunction to release cytochrome C into the cytoplasm and activate the caspase cascade and apoptosis stimulation [83, 84]. As a result of this investigation and previous articles, we believe that paclitaxel-induced renal and cardiac dysfunction and histopathology are caused by increased oxidative stress and attenuation of antioxidant defenses.

Paclitaxel-induced oxidative stress in the kidney or heart was remarkably suppressed by rutin and hesperidin treatment with reduced LPO and elevated GSH content and activities of antioxidant enzymes. Both compounds enhance endogenous antioxidant activity beyond their ability to scavenge free radicals and reduce the formation of lipid peroxide radicals. Remarkably similar results were mentioned by Geetha et al. [85], Huang et al. [86], and Xianchu et al. [76], who found that rutin suppressed oxidative stress *via* lowering production of ROS and MDA and by augmenting antioxidant status through increasing SOD, GSH, and GPx in several models of cardiovascular disease. An article of Qu et al. [27] showed that rutin suppressed oxidative/nitrosative stress, inflammation, and apoptosis in rats' kidneys. Rutin exhibited a significant level of protection against acrylamide-induced oxidative DNA damage, likely due to its antioxidant property [87].

By alleviating the oxidative stress, endoplasmic reticulum stress, inflammation, apoptosis, and autophagy-induced by valproic acid, rutin administration ameliorated liver and kidney damage [88]. It was found that the protective effects of hesperidin were associated with countering oxidative/nitrosative stress, inflammation, and apoptosis, thus, preserving renal structure and function in mice intoxicated with cyclophosphamide [89]. Moreover, hesperidin reduced the heart LPO and increased antioxidant enzyme activities in ischemic myocardial rats [90]. Hesperidin's anticancer potential is controlled by ROS-dependent apoptotic pathways in certain cancer cells, despite the fact that it can be an excellent ROS scavenger and could operate as a powerful antioxidant defense mechanism [91, 92].

## 5. Conclusion

Coadministration of rutin, hesperidin, or their combination with paclitaxel in male Wistar rats might diminish the incidence and severity of detrimental effects of paclitaxel (Taxol)-induced toxicity in the kidney and heart. These protective effects are likely mediated by suppressing oxidative stress and enhancing antioxidant defenses. Moreover, a combination of rutin and hesperidin treatment of paclitaxel-administered rats was most efficacious in preventing renal and cardiac dysfunction and adverse histological impacts. Before using rutin and hesperidin in patients and receiving FDA approval, more clinical trials are required to evaluate their effectiveness and safety.

## Figures and Tables

**Table 1:** Correlation between the kidney function biomarkers and oxidative stress bioindicators.

	Creatinine		Urea		Uric acid	
	*r*	*p* value	*r*	*p* value	*r*	*p* value
LPO	0.633	*p* < 0.001	0.457	*p* < 0.05	0.721	*p* < 0.001
GSH	−0.588	*p* < 0.01	−0.489	*p* < 0.01	−0.780	*p* < 0.001
SOD	−0.601	*p* < 0.001	−0.542	*p* < 0.01	−0.686	*p* < 0.001
GPx	−0.570	*p* < 0.01	−0.536	*p* < 0.01	−0.623	*p* < 0.001

*r*: correlation value. *p* > 0.05, nonsignificant; significance was calculated at these levels: *p* < 0.05, *p* < 0.01, and *p* < 0.001.

**Table 2:** Correlation between the heart function biomarkers and oxidative stress bioindicators.

	CK-MB		LDH	
	*r*	*p* value	*r*	*p* value
LPO	0.664	*p* < 0.001	0.705	*p* < 0.001
GSH	−0.589	*p* < 0.01	−0.421	*p* < 0.05
SOD	−0.645	*p* < 0.001	−0.600	*p* < 0.001
GPx	−0.607	*p* < 0.001	−0.504	*p* < 0.01

*r*: correlation value. *p* > 0.05, nonsignificant; significance was calculated at these levels: *p* < 0.05, *p* < 0.01, and *p* < 0.001.

**Table 3:** Pathological renal lesion scores in different groups.

Groups	Parameters				
	Degenerative renal tubules	Necrosis of renal tubules	Congestion	Leucocytic infiltration	Glomerulonephrosis
Normal	−	−	−	−	−
Paclitaxel	++	++	+++	+++	+++
Paclitaxel + rutin	++	−	++	++	++
Paclitaxel + hesperidin	++	+	++	++	+++
Paclitaxel + rutin + hesperidin	++	+	+++	+	++

Lesion types are (−) absence, (+) minimal, (++) mild, (+++) moderate, and (++++) severe.

**Table 4:** Pathological cardiac lesion scores in different groups.

Groups	Parameters	
	Coagulative necrosis (hyalinosis)	Leucocytic infiltration
Normal	−	−
Paclitaxel	++	+++
Paclitaxel + rutin	+	+
Paclitaxel + hesperidin	++	−
Paclitaxel + rutin + hesperidin	+	−

Lesion types are (−) absence, (+) minimal, (++) mild, (+++) moderate, and (++++) severe.

## Data Availability

All data are available from the corresponding author upon reasonable request.

## Abbreviations

ANOVA: One-way analysis of variance
AST: Aspartate aminotransferase
CK: Creatine kinase
CK-MB: Creatine kinase-MB
CMC: Carboxymethylcellulose
CrEL: Cremophor/ethanol
DNA: Deoxyribonucleic acid
FDA: Food and Drug Administration
GPx: Glutathione peroxidase
GSH: Reduced glutathione
GSSG: Oxidized glutathione
H&E: Hematoxylin and eosin stain
H_2_O_2_: Hydrogen peroxide
b. wt: Body weight
LDH: Lactate dehydrogenase
LPO: Lipid peroxidation
MDA: Malondialdehyde or 1,1,3,3-tetramethoxypropane
rpm: Round per minute
ROS: Reactive oxygen species
SEM: Standard error of the mean
SPSS: Statistical package for social sciences
SOD: Superoxide dismutase
TBA: Thiobarbituric acid
TCA: Trichloroacetic acid.
